# Grapevine Shoot Tip Cryopreservation and Cryotherapy: Secure Storage of Disease-Free Plants

**DOI:** 10.3390/plants10102190

**Published:** 2021-10-15

**Authors:** Jean Carlos Bettoni, Zvjezdana Marković, Wenlu Bi, Gayle M. Volk, Toshikazu Matsumoto, Qiao-Chun Wang

**Affiliations:** 1 The New Zealand Institute for Plant and Food Research Limited, Batchelar Road, Palmerston North 4410, New Zealand; 2 Department of Viticulture and Enology, Faculty of Agriculture, University of Zagreb, Svetišimunska Cesta 25, 10000 Zagreb, Croatia; zmarkovic@agr.hr; 3 Department of Plant Agriculture, Gosling Research Institute for Plant Preservation, University of Guelph, Guelph, ON N1G 2W1, Canada; wenlubi@uoguelph.ca; 4 USDA-ARS National Laboratory for Genetic Resources Preservation, 1111 S. Mason Street, Fort Collins, CO 80521, USA; Gayle.Volk@ars.usda.gov; 5 Department of Agricultural and Forest Sciences, Faculty of Life and Environmental Science, Shimane University, 1060 Nishi-Kawatsucho, Matsue 690-8504, Japan; tmatsumoto@life.shimane-u.ac.jp; 6 State Key Laboratory of Crop Stress Biology in Arid Region, College of Horticulture, Northwest A&F University, Yangling District, Shaanxi 712100, China; qiaochunwang@nwsuaf.edu.cn

**Keywords:** cryo-banks, virus-free material, ex situ conservation, plant vitrification solution, micropropagation, *Vitis*

## Abstract

Grapevine (*Vitis* spp.) is one of the most economically important temperate fruit crops. Grapevine breeding programs require access to high-quality *Vitis* cultivars and wild species, which may be maintained within genebanks. Shoot tip cryopreservation is a valuable technique for the safe, long-term conservation of *Vitis* genetic resources that complements traditional field and in vitro germplasm collections. *Vitis* is highly susceptible to virus infections. Virus-free plants are required as propagation material for clonally propagated germplasm, and also for the global exchange of grapevine genetic resources. Shoot tip cryotherapy, a method based on cryopreservation, has proven to be effective in eradicating viruses from infected plants, including grapevine. This comprehensive review outlines/documents the advances in *Vitis* shoot tip cryopreservation and cryotherapy that have resulted in healthy plants with high regrowth levels across diverse *Vitis* species.

## 1. Introduction

Grapevines (*Vitis* spp.) are among the most important fruit crops worldwide, with plantings still expanding. Globally, the total grape-growing acreage reached about 7.4 million ha in 2019 [1]. *Vitis vinifera* is the major cultivated species, with about 5000 cultivars available worldwide [2,3]. An extensive grape market benefits from the continuous breeding of elite cultivars. The *Vitis* genus has about 80 species, and some species, such as those native to North America, Chinese wild species, and *Muscadinia rotundifolia*, contain valuable genes or traits that make them resistant/tolerant to abiotic and biotic stresses, and can be used for breeding elite cultivars [4,5,6,7,8,9,10]; for example, *V. pseudoreticulata* ‘Baihe-35-1’ (a Chinese wild species) [8], *V. aestivalis* ‘Norton’ [9], and *M. rotundifolia* ‘Regale’ [10] are highly resistant to powdery mildew caused by *Erysiphe necator*. Therefore, it is necessary to preserve *Vitis* diverse genetic resources to ensure continued access to potentially valuable traits [6,11].

Grapevine breeding programs require access to high-quality *Vitis* cultivars and wild species, which may be maintained within genebanks. Ideally, these genebank collections have been characterized using molecular markers, evaluated with standardized phenotyping methods, and documented in public databases [11]. Traditionally, *Vitis* genebanks are maintained as whole plants in field collections and/or as stock cultures in in vitro culture [12,13]; for example, the US Department of Agriculture-Agricultural Research Service (USDA-ARS) National Clonal Germplasm Repository for Tree Fruit and Nut Crops and Grapes in Davis (CA, USA) maintains one of the most diverse *Vitis* collections in the world (42 taxa), where 3649 unique accessions of grapes are maintained in duplicate as vines in the field [14].

Field genebanks allow inventories to be observed throughout the year, and provide readily available plant material for use in breeding programs and propagation. However, they are expensive and high maintenance due to their intensive management requirements, and are at risk of losses from attacks by pests and diseases, and environmental disasters [15,16,17]. In addition to the large range of phenologies and genetic diversity, accessions also respond differently to cultural treatments, spray regimens, and pathogen/disease threats. The establishment of duplicate collections in a secondary field site is a commendable strategy to minimize the risk of loss; however, it adds to the expense of maintenance, and the collections could still be threatened by pathogens and pests [16,17]. In vitro genebanks provide an alternative to field collections for the short- and medium-term storage of *Vitis* germplasm [12,18,19,20,21]. In addition, they can be a source of clean plants for other purposes, including as source plant material for cryopreservation. However, in vitro genebanks are also labor intensive, plus they have risks of microbial contamination and genetic or somaclonal variation [22,23,24,25,26].

Reliable and robust back-up methods are required to augment the labor-intensive maintenance of field and in vitro collections. Cryopreservation, the storage of biological materials in liquid nitrogen (LN, −196 °C) or in its vapor phase (LNV, approx. −185 °C), is now being considered as the most safe and cost-effective strategy for the long-term storage of plant genetic resources [15,27]. It complements traditional field genebanks and in vitro collections, and overcomes the above-mentioned limitations. Under cryopreserved conditions, plant material is preserved in a state whereby cellular divisions and metabolic processes are minimized, thus preserving the genetic integrity for a longer duration, without any manipulations [15,22,27,28,29]. Although reliable access to LN is necessary, overall, these collections require minimal space and maintenance. They also minimize the risk of biotic threats compared to field or in vitro collections [15,22].

Grapevines are genetically highly heterozygous, thus vegetative propagules, such as shoot tips and dormant buds, are the most suitable propagule source for the clonal preservation of cultivars in *Vitis* germplasm collections. The cryopreservation of dormant buds could be a potentially good approach, but attempts in a few *Vitis* species have been mostly unsuccessful [30]. Shoot tips are by far the most utilized explants for *Vitis* cryopreservation [16,31,32,33,34,35,36,37]. Shoot tips are tissues that include the apical dome (AD) and a number of leaf primordia (LP), and are able to regenerate entire plants. In some cases, as in wild species representatives of *Vitis* germplasm, other conservation targets, such as pollen [38,39], seeds [40], and somatic embryos [41,42,43,44,45], may be of interest [46,47].

*Vitis* is highly susceptible to virus infections. The vegetative propagation of grapevines has resulted in virus transmission from generation to generation, and virus titers have accumulated as a result of repeated propagation events [48,49,50]. Along with inducing increased susceptibility to other pathogens, viral diseases can cause economic losses as a result of their negative effects on yield and quality [50]. The cultivation of virus-free plants is needed to successfully control viral diseases and for the global exchange of genetic resources [51,52,53]. Based on cryopreservation techniques, shoot tip cryotherapy has proven to be effective in eradicating virus infections from infected plants [54,55,56,57,58,59,60,61], including grapevine [34,51,52,62,63,64,65,66,67].

Cryopreservation procedures have been established and implemented for many vegetatively propagated genebank collections [22,29,68,69,70,71,72,73,74]. Cryopreservation protocols have been described for grapevines, dating back to as early as the 1990s (Table 1); however, *Vitis* cryo-storage has been challenging and has not been widely used within genebanks. The recent advances in grapevine cryopreservation procedures, applicable to a wide range of *Vitis* species, may overcome the genotype-specific responses that have posed some of the major challenges in implementing *Vitis* cryopreservation procedures in genebanks [35,36,37,75,76]. This review provides updated and comprehensive information on the development and recent progress of *Vitis* shoot tip cryopreservation and cryotherapy that resulted in healthy plants with high regrowth levels across diverse *Vitis* species.

## 2. Explant Sources

The cryopreservation of shoot tips sourced from in vitro-grown plants is a standard procedure in many laboratories [29,67,77]. The source plant age, physiological state and growth phase, and quality of the in vitro stock cultures and shoot tips play significant roles in successful cryoprocedures [27,77,78,79].

Regardless of the explant sources, all shoot tip cryopreservation protocols use tissue culture to some extent. Therefore, the first essential step, before considering cryopreservation, is to optimize the micropropagation system, ensuring that optimal culture media formulation and growth conditions have been established to induce favorable physiological conditions for shoot tip donor plants [46,80,81].

**Figure 1:**
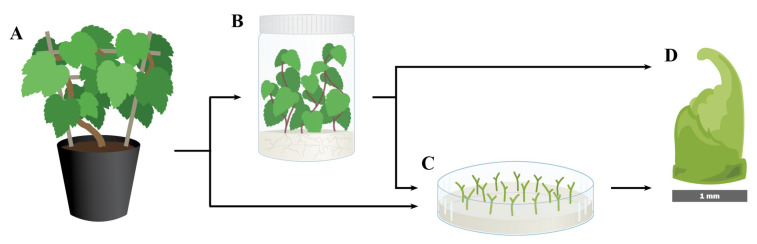
Production and preparation of grapevine shoot tips for cryopreservation procedures. Nodal sections (micro-cuttings) sourced from either greenhouse-grown plants (**A**) or in vitro stock cultures (**B**), either with or without a two-week culture in shooting medium (**C**,**D**) prior to excision of apical shoot tips (**D**).

Shoot tips excised from apical or axillary buds of in vitro-grown cultures are usually the most common explant used for the cryopreservation of vegetatively propagated species, including grapevine (Table 1; Figure 1B,C). When available, in vitro stock cultures provide a source of material for cryopreservation throughout the year, and they are easy to multiply and to manipulate [82,83]. It is preferable to introduce plants into tissue culture from actively growing shoots to establish clean in vitro stock cultures. However, the process of introducing plants into tissue culture, and the subsequent multiplication steps dramatically increase the cost, labor and time requirements of the cryopreservation procedure [46,67]. It could take a period of six to twelve months to introduce a *Vitis* accession into tissue culture and produce adequate amounts of culture for cryopreservation.

**Table 1:** List of research reported from 1989 to 2021 on grapevine shoot tip cryopreservation.

Species, No. Genotypes Tested	Explant Source	Cryo Method	Pretreatment	Preculture	Best Cryoprotectant/ Dehydration Treatment	Regrowth (%)	Year Ref.
*V. labrusca*, 3	ST (1–2 mm; type n/s) harvested from greenhouse plants	TEC	None	None	10% DMSO + 60 g L^−1^ sucrose (2 h at 20 °C) → cooled (0.5 °C min^−1^) to −20 °C/−30 °C/−40 °C → LN	87–100% survival	1989 [84]
*V. vinifera*, 1	AxST (size n/s) harvested from in vitro cultures that are 7 weeks old	ED + TEC	None	Liquid MS with increased sucrose concentrations every 2 d of 0.3, 0.5 and 0.75 M every 2 d, and then every 1 day of 1, 1.25 and 1.5 M (temperature n/s)	Bead desiccation to 20% + cooled (0.5 °C min^−1^) from +20 °C to −80 °C → LN	24	1991 [85]
		ED			Bead desiccation to 20% → LN	No shoot regrowth	
*V. vinifera*, 1	AxST (size n/s) harvested from in vitro cultures that are 7 to 8 weeks old	ED + TEC	None	Liquid MS with increased sucrose concentrations every 2 days of 0.3, 0.5 and 0.75 M and 1 M (temperature n/s)	Bead desiccation to 22% + cooled (0.5 °C min^−1^) from + 20 °C to −100 °C → LN	30	1993 [86]
		ED			Bead desiccation to 22% → LN	30% survival	
*V. vinifera*, 3	AxST (size n/s) harvested from in vitro cultures (age n/s)	ED + TEC	None	Liquid MS with increased sucrose concentrations every 24 h of 0.3, 0.6 and 1 M (25 °C)	Bead desiccation to 30% + beads cooled from 0.5 °C min^−1^ to −80 °C → LN	No shoot regrowth	2000 [87]
*LN33* (*Vitis* L.), 1; *V. vinifera*, 1	ST (1 mm; type n/s) harvested from in vitro cultures that are 4 weeks old	ED	None	1/2 MS with increased sucrose concentrations every 24 h of 0.25, 0.5, 0.75 and 1 M + 2.6 g L^−1^ gellan gum (24 °C)	Bead desiccation to 16% → LN	40–60% survival	2000 [88]
*V. vinifera*, 4	AxST (2 mm) harvested from in vitro cultures that are 5 months old	ED + TEC	Cold-hardening for 4 weeks at 5 °C	B5 medium with increased sucrose concentrations every 24 h of 0.1, 0.3, 0.7 and 1 M + 5 g L^−1^ agar (5 °C)	Bead desiccation to 21% + cooled (0.2 °C min^−1^) to −40 °C → LN	15–40	2001 [89]
**Table 1** (continued)							
**Species, No. Genotypes Tested**	**Explant Source**	**Cryo** **Method**	**Pretreatment**	**Preculture**	**Best Cryoprotectant/** **Dehydration Treatment**	**Regrowth (%)**	**Year** **Ref.**
*LN33* (*Vitis* L.), 1	ST (1 mm; type n/s) harvested from in vitro cultures that are 4 weeks old	VI	None	1/2 MS with increased sucrose concentrations every 24 h of 0.25, 0.5 and 0.75 M + 2.6 g L^−1^ Gelrite (24 °C)	2 M glycerol + 0.75 M sucrose (60 min at 25 °C) → 1/2 PVS2 (30 min at 0 °C) → PVS2 (50 min at 0 °C) → LN	45% survival	2003 [33]
		ED		Preculture described above + additional 1 d on 1 M sucrose + 2.6 g L^−1^ Gelrite	Bead desiccation by air-drying for 8 h (beads moisture content n/s) → LN	63% survival	
*V. vinifera*, 1	ST (1 mm; type n/s) harvested from in vitro cultures that are 4 weeks old	VI	None	1/2 MS with increased sucrose concentrations every 24 h of 0.25, 0.5 and 0.75 M + 2.6 g L^−1^ Gelrite (24 °C)	2 M glycerol + 0.75 M sucrose (60 min at 25 °C) → 1/2 PVS2 (30 min at 0 °C) → PVS2 (50 min at 0 °C) → LN	50% survival	2003 [64]
		ED		Preculture described above + additional 1 day on 1 M sucrose + 2.6 g L^−1^ Gelrite	Bead desiccation by air-drying for 7 h (beads moisture content n/s) → LN	62% survival	
*V. berlandieri x V. riparia*, 1	AST and AxST (1–2 mm) harvested from in vitro cultures (age n/s)	EN-VI	Cold-hardening for 3 weeks at 4 °C	None	PVS2 (30 and 90 min at 0 °C) → LN	Low (n/s)	2003 [90]
*V. vinifera*, 7; *V. berlandieri* x *riparia*, 2; *V. mourvedre* × *V. rupestris*, 1; *V. coignetiae*, 1	AxST (1 mm) harvested from in vitro cultures that are 4 to 5 months old	VI	None	1/2 MS + 0.3 M sucrose + 2 g L^−1^ gellan gum for 3 days at 25 °C	2 M glycerol + 0.4 M sucrose (20 min at 25 °C) → 1/2 PVS2 (30 min at 0 °C) → PVS2 (50 min at 0 °C) → LN	30–87	2000 [31] 2003 [32]
*V. vinifera*, 4	AST (2 mm) harvested from in vitro cultures that are 50 days old	ED + TEC	None	Culture medium with increased sucrose concentrations every 24 h of 0.5, 0.70 and 1 M (5 °C)	Bead desiccation to 26% + cooled (0.2 °C min^−1^) from 0 °C to −40 °C → LN	36 (average)	2003 [91]
*V. berlandieri* × *riparia*, 1	AxST (size n/s) harvested from in vitro cultures (age n/s)	VI	None	0.2, 0.3 or 0.4 M sucrose (duration and conditions n/s)	2 M glycerol + 0.4 M sucrose (30 min) → PVS2 (30, 60 or 90 min) (conditions n/s) → LN	No shoot regrowth	2007 [92]
**Table 1** (continued)							
**Species, No. Genotypes Tested**	**Explant Source**	**Cryo** **Method**	**Pretreatment**	**Preculture**	**Best Cryoprotectant/** **Dehydration Treatment**	**Regrowth (%)**	**Year** **Ref.**
*V. vinifera*, 1	ST (1 mm; type n/s) harvested from shoots of greenhouse plants	ED	None	3/4 MS with increased sucrose concentrations every 24 h of 0.25, 0.5, 0.75 and 1 M (conditions n/s)	Bead desiccation by air-drying for 12 h (beads moisture content n/s) → LN	59	2011 [63]
*V. vinifera*, 1	ST (2–3 mm; type n/s) harvested from in vitro cultures (age n/s)	VI	None	MS + 0.3 M sucrose + 8 g L^−1^ agar for 1 day at 24 °C	5% (*w*/*v*) DMSO + 5% (*w*/*v*) glycerol + 5% (*w*/*v*) sucrose (50 min at 0 °C) → PVS2 (40 min at 0 °C) or MPVS2 **** (40 min at 0 °C) → LN	47 and 55	2011 [93]
*V. berlandieri* × *V. riparia*, 1	AxST (2 ± 1 mm) harvested from in vitro cultures (age n/s)	VI	Cold-hardening for 2 weeks at 4 °C	MS + 0.2, 0.3 or 0.4 M sucrose + 8 g L^−1^ agar for 2 days at 4 °C	2 M glycerol + 0.4 M sucrose (30 min at 4 °C) → PVS2 (30, 60 or 90 min at 0 °C) → LN	No shoot regrowth	2012 [94]
*V. vinifera*, 1	AST (1 mm) harvested from the lateral shoots of in vitro nodal sections cultured for 2 weeks	ED	Nodal sections on 1/2 MS + Morel’s vitamins * + 20 g L^−1^ sucrose + 1 μmol ZR + 7 g L^−1^ agar for 2 weeks at 24 °C	Liquid 1/2 MS with increased sucrose concentrations every 12 h of 0.25, 0.5, 0.75 and 1 M (24 °C)	Bead desiccation to 22.3% → LN	37	2013 [95]
		DV		MS + 1 M sucrose + 7 g L^−1^ agar for 24 h at 24 °C	2 M glycerol + 0.4 M sucrose (20 min at RT) → PVS2 (50 min at 0 °C) → LN	50	
*V. vinifera*, 2	AxST (1 mm) harvested from greenhouse-grown plants	DV	None	1/2 MS + 0.3 M sucrose + 0.16 mM GSH reduced + 0.14 mM AsA + 2.5 g L^−1^ gellan gum for 3 days at 25 °C	2 M glycerol + 0.4 M sucrose (20 min at 22 °C) → 1/2 PVS2 (10–15 min at 0 °C) → PVS2 (10–20 min at 0 °C) → LN	40–46	2013 [96]
**Table 1** (continued)							
**Species, No. Genotypes Tested**	**Explant Source**	**Cryo** **Method**	**Pretreatment**	**Preculture**	**Best Cryoprotectant/** **Dehydration Treatment**	**Regrowth (%)**	**Year** **Ref.**
*V. vinifera*, 1	AST (1 mm) harvested from the lateral shoots of in vitro nodal sections cultured for 2 weeks	DV	Nodal sections on 1/2 MS + Morel’s vitamins * + 20 g L^−1^ sucrose + 1 μmol BA or ZR + 7 g L^−1^ agar for 2 weeks at 24 °C	1/2 MS + 0.1 M sucrose + 7 g L^−1^ agar for 24 h at 25 °C	2 M glycerol + 0.4 M sucrose (20 min at RT) → 1/2 PVS2 (30 min at RT) → PVS2 (50 min at 0 °C) → LN	44	2014 [97]
	AxST (1 mm) harvested from in vitro cultures that are 2 months old		None		2 M glycerol + 0.4 M sucrose (20 min at RT) → 1/2 PVS2 (30 min at RT) → PVS2 (75 min at 0 °C) → LN	41.6	
*V. vinifera*, 1	AxST (size n/s) harvested from vineyard	VI	None	None	PVS2 (180 min at 25 °C) → LN	n/s	2015 [98]
*V. vinifera*, 9	AST (1 mm) harvested from the lateral shoots of in vitro nodal sections cultured for 2 weeks	DV	Nodal sections on 1/2 MS + Morel’s vitamins * + 20 g L^−1^ sucrose + 1 μmol BA + 7 g L^−1^ agar for 2 weeks at 24 °C	1/2 MS + 0.1 M sucrose + 7 g L^−1^ agar for 24 h at 25 °C	2 M glycerol + 0.4 M sucrose (20 min at RT) → 1/2 PVS2 (30 min at RT) → PVS2 (50 min at 0 °C) → LN	0–70	2015 [34]
*V. vinifera*, 7; *V. labrusca*, 1; *V. riparia*, 1; *V. berlandieri* × *V. rupestris*, 2; *V. berlandieri* × *V. riparia*, 1	AST harvest from in vitro cultures (size and age of cultures n/s)	ED	None	Following Wang et al. [32]		0–9% survival	2015 [99]
		VI	None	Following Shatnawi et al. [83]		0–1% survival	
*V. vinifera*, 5	AST and AxST harvested from in vitro cultures that are 2 weeks old (size n/s)	DV	Cultures on 1/2 MS + B5 vitamins ** + 20 g L^−1^ sucrose + 0.5 mg L^−1^ BA + 0.1 mM SA + 3 g L^−1^ gellan gum for 2 weeks at 24 °C	1/2 MS + B5 vitamins ** with increased sucrose concentrations of 0.25 M, 0.5 M, 0.75 M and 1 M (every 24 h) + 3 g L^−1^ Gelrite (24 °C)	2 M glycerol + 0.4 M sucrose (20 min at RT) → PVS2 (36–41.5 min at 0 °C) → LN	13–30	2015 [62]
**Table 1** (continued)							
**Species, No. Genotypes Tested**	**Explant Source**	**Cryo** **Method**	**Pretreatment**	**Preculture**	**Best Cryoprotectant/** **Dehydration Treatment**	**Regrowth (%)**	**Year** **Ref.**
*V. vinifera*, 3	AST (1–2 mm) harvested from the lateral shoots of in vitro nodal sections cultured for 10 days	VI	None	Medium with increased sucrose concentrations of 0.3, 0.5 and 0.75 M every 24 h (conditions n/s)	2 M glycerol + 0.4 M sucrose (30 min at RT) → 2 M glycerol + 0.75 M sucrose (30 min at RT) → 1/2 PVS3 (30 min at RT) → 80 % PVS3 (60–90 min)	n/s after LN exposure	2015 [100]
*V. vinifera*, 4; *V. riparia* × *V. rupestris*, 1; *V. vinifera Chasselas* × *V. berlandieri*, 1	AST and AxST (1–1.5 mm) harvested from the lateral shoots of in vitro nodal sections cultured for 2 weeks	DV	Nodal sections on 1/2 MS + B5 vitamins ** + 20 g L^−1^ sucrose + 0.5 mg L^−1^ BA + 0.1 Mm SA + 3 g L^−1^ gellan gum for 2 weeks at 24 °C	1/2 MS + B5 vitamins ** with increased sucrose concentrations every 24 h of 0.25 M, 0.5 M, 0.75 M and 1 M + 3 g L^−1^ Gelrite (24 °C)	2 M glycerol + 0.4 M sucrose (20 min at RT) → PVS2 (36–43 min at 0 °C) → LN	7–45	2016 [16]
*V. vinifera*, 6; *V. pseudoreticulata*, 2	AST (1 mm) harvested from the lateral shoots of in vitro nodal sections cultured for 2 weeks	DV	None	1/2 MS + 0.3 M sucrose + 0.16 mM GSH + 0.14 mM AsA + 7 g L^−1^ agar for 3 days at 24 °C	2 M glycerol + 0.4 M sucrose (20 min at 24 °C) → 1/2 PVS2 (30 min at 0 °C) → PVS2 (50 min at 0 °C) → LN	24–72	2018 [35]
*V. vinifera*, 2; *V. vinifera* × *V. labrusca*, 1; *V. pseudoreticulata*, 1						43–59	2018 [51]
*V. vinifera*, 1; *V. aestivalis*, 1; *V. afghanistan*, 1; *V. flexuosa*, 1; *V. palmate*, 1; *V. riparia*, 1; *V. rupestris*, 1; *V. sylvestris*, 1; *V. treleasii*, 1	AST (1–1.5 mm) harvested from the lateral shoots of in vitro nodal sections cultured for 2 to 3 weeks	DV	Nodal sections on MS + 30 g L^−1^ sucrose + 0.2 mg L^−1^ BA + 0.1 mM SA + 1 mM GSH*** + 1 mM AsA + 3 g L^−1^ gellan gum for 2–3 weeks at 25 °C	1/2 MS + 0.3 M sucrose + 0.1 mM SA + 1 mM GSH *** + 1 mM AsA + 3 g L^−1^ gellan gum for 3 days at 25 °C	2 M glycerol + 0.4 M sucrose (20 min at 22 °C) → 1/2 PVS2 (30 min at 22 °C) → PVS2 (90 min at 0 °C) → LN	25–43	2018 [36]
**Table 1** (continued)							
**Species, No. Genotypes Tested**	**Explant Source**	**Cryo** **Method**	**Pretreatment**	**Preculture**	**Best Cryoprotectant/** **Dehydration Treatment**	**Regrowth (%)**	**Year** **Ref.**
*V. champinii* × 1613 Couderc, 1; *V. berlandieri* × *V. riparia*, 1; *V. shampinii*, 1	ST (3 mm; type n/s) harvested from shoots of greenhouse plants	DV	None	1/2 MS + 0.3 M sucrose for 3 days at 25 °C	2 M glycerol + 0.4 M sucrose (20 min at 25 °C) → PVS2 (0–50 min at 0 °C) → LN	No shoot regrowth	2019 [101]
				1/2 MS with increased sucrose concentrations every 24 h of 0.25, 0.5, 0.75 and 1 M (25 °C)	2 M glycerol + 0.4 M sucrose (20 min at 25 °C) → PVS2 (50 min at 0 °C) → LN	27–47	
*V. vinifera*, 2; *V. berlandieri* × *V. riparia*, 1	AST (1 mm) harvested from the lateral shoots of nodal sections cultured for 2 weeks from growth chamber stock plants	DV	Nodal sections on MS + 30 g L^−1^ sucrose + 0.2 mg L^−1^ BA + 0.1 mM SA + 1 mM GSH *** + 1 mM AsA + 1.5 % (*v*/*v*) PPM + 3 g L^−1^ gellan gum for 2 weeks at 25 °C	1/2 MS + 0.3 M sucrose + 0.1 mM SA + 1 mM GSH *** + 1 mM AsA + 1.5 % (*v*/*v*) PPM + 3 g L^−1^ gellan gum for 3 days at 25 °C	2 M glycerol + 0.4 M sucrose (20 min at 22 °C) → 1/2 PVS2 (30 min at 22 °C) → PVS2 (30–40 min at 0 °C) → LN	43–64	2019 [102]
*V. vinifera*, 2; *V. actinifolia*, 1; *V. aestivalis*, 1; *V. jacquemontii*, 1; *V. flexuosa*, 1; *V. palmate*, 1; *V. riparia*, 1; *V. rupestris*, 1; *V. sylvestris*, 1; *V. ficifolia*, 1; *V. treleasi*, 1; *V. xnovae angeliae*, 1	AST (1 mm) harvested from the lateral shoots of in vitro nodal sections cultured for 2 weeks	DV	Nodal sections on MS + 30 g L^−1^ sucrose + 0.2 mg L^−1^ BA + 0.1 mM SA + 1 mM GSH *** + 1 mM AsA + 3 g L^−1^ gellan gum for 2 weeks at 25 °C	1/2 MS + 0.3 M sucrose + 0.1 Mm SA + 1 mM GSH *** + 1 mM AsA + 3 g L^−1^ gellan gum for 3 days at 25 °C	2 M glycerol + 0.4 M sucrose (20 min at 22 °C) → 1/2 PVS2 (30 min at 22 °C) → PVS2 (90 min at 0 °C) → LN	35–72	2019 [37]
			Pretreatment described above + cold-hardening for 2 weeks at 5 °C		2 M glycerol + 0.4 M sucrose (20 min at 22 °C) → 1/2 PVS2 (30 min at 22 °C) → PVS2 (75 min at 0 °C) → LN	43–70	
*V. aestivalis*, 1; *V. jacquemontii,* 1	AST (1 mm) harvested from the lateral shoots of in vitro nodal sections cultured for 2 weeks	DV	Nodal sections on MS + 30 g L^−1^ sucrose + 0.2 mg L^−1^ BA + 0.1 mM SA + 1 mM GSH *** + 1 mM AsA + 3 g L^−1^ gellan gum for 2 weeks at 25 °C	1/2 MS + 0.3 M sucrose + 0.1 mM SA + 1 mM GSH *** + 1 mM AsA + 3 g L^−1^ gellan gum for 3 days at 25 °C	2 M glycerol + 0.4 M sucrose (20 min at 22 °C) → 1/2 PVS2 (30 min at 22 °C) → PVS2 (90 min at 0 °C) → LN	53–70	2019 [76]
		V-CP			2 M glycerol + 0.4 M sucrose (30 min at 22 °C) → PVS2 (40 min at 22 °C) → LN	68–70	
**Table 1** (continued)							
**Species, No. Genotypes Tested**	**Explant Source**	**Cryo** **Method**	**Pretreatment**	**Preculture**	**Best Cryoprotectant/** **Dehydration Treatment**	**Regrowth (%)**	**Year** **Ref.**
*V. vinifera*, 1	AST (1 mm) harvested from the lateral shoots of in vitro nodal sections cultured for 2 to 3 weeks	DV	Nodal sections on MS + 30 g L^−1^ sucrose + 0.2 mg L^−1^ BA + 0.1 mM SA + 1 mM GSH *** + 1 mM AsA + 3 g L^−1^ gellan gum for 2–3 weeks at 25 °C	1/2 MS + 0.3 M sucrose + 0.1 mM SA + 1 mM GSH *** + 1 mM AsA + 3 g L^−1^ gellan gum for 3 days at 25 °C	2 M glycerol + 0.4 M sucrose (30 min at 22 °C) → 1/2 PVS2 (30 min at 22 °C) → PVS2 (75 min at 0 °C) → LN	68	2019 [103]
	AST (1 mm) harvested from growth chamber nodal sections cultured in vitro for 2 weeks before ST excision		Pretreatment described above + additional + 1.5% (*v*/*v*) PPM for 2 weeks at 25 °C	Preculture described above + additional + 1.5% (*v*/*v*) PPM for 3 days at 25 °C	2 M glycerol + 0.4 M sucrose (30 min at 22 °C) → 1/2 PVS2 (30 min at 22 °C) → PVS2 (40 min at 0 °C) → LN	43	
*V. vinifera*, 1	ST (size and type n/s) harvested from in vitro stock cultures that are 4 weeks old	ED	Cold-hardening for 4 weeks at 5 °C	1/2 MS with increased sucrose concentrations every 24 h of 0.25, 0.5, 0.75 and 1 M + 2.5 g L^−1^ Phytagel (5 °C)	Bead desiccation to 18.4% → LN	33% survival	2021 [104]

* Morel et al. [105]; ** Gamborg et al. [106]; *** reduced form; **** MPVS2: 15% (*w*/*v*) glycerol, 20% (*w*/*v*) DMSO, 15% ethylene glycol + 14% (*w*/*v*) sucrose in full-strength MS medium. ST: shoot tips; AxST: axillary shoot tips; AST: apical shoot tips; TEC: two-step cooling; ED: encapsulation-dehydration; VI: vitrification; EN-VI: encapsulation-vitrification; DV: droplet-vitrification; V-CP: V cryo-plate; SA: salicylic acid; AsA: ascorbic acid; GSH: glutathione; ZR: zeatin riboside; BA: benzylaminopurine; PVS2: plant vitrification solution 2; PVS3: plant vitrification solution 3; PPM: plant preservative mixture; n/s: not specified; RT: room temperature.

Recently, two studies reported the cryopreservation of grapevine shoot tips that were collected directly from greenhouse-grown plants [96,102]. Hassan and Haggag [96] simplified the cryoprocedure by sampling shoot tips directly from greenhouse-grown plants, and reported regrowth percentages of 40% for *V. vinifera* ‘Black Matrouh’ and 47% for ‘Bez El-Anza’ after LN exposure. One limitation with this method is that shoot tips harvested directly from greenhouse-grown plants have non-uniform developmental stages and, thus, can exhibit highly variable responses to cryoexposure. Bettoni et al. [102] focused on improving the shoot tip quality and uniformity, plus reducing the effects of oxidation and microbial contamination by excising shoot tips from pretreated nodal sections harvested from greenhouse-grown plants (Figure 1A,C,D). With these optimized parameters, values of 43, 64 and 48% shoot regrowth after LN exposure were obtained for *V. vinifera* cvs. Chardonnay and Riesling, and the rootstock selection ‘Oppenheim #4’ (*V*. *berlandieri* × *V. riparia*), respectively. Cryopreservation protocols that make use of shoot tips derived from ex vitro sourced plants require less manipulation than those from in vitro stock cultures, increasing the efficiency of cryoprocessing in genebanks by significantly reducing the laborious steps of in vitro culture establishment and multiplication. Research suggests that it may be possible to cryopreserve *Vitis* shoot tips without introducing each accession into tissue culture first. However, this strategy can only be applied when ex vitro materials can be easily disinfected prior to shoot tip excision [102,107].

## 3. Pretreatment, Excision and Preculture of Shoot Tips

Pretreatment, to induce tolerance to dehydration and subsequent freezing in LN, is necessary for the successful cryopreservation of shoot tips [16,35,36,37,97]. The optimal pretreatments of in vitro stock shoots can differ among species, and may include cold-hardening [89,108,109,110,111], exogenous application of osmotic agents, such as sorbitol, mannitol, and sugars, antioxidants, such as glutathione (GSH) and ascorbic acid (AsA) [35,36,37,76,108,112], polyvinylpyrrolidone [113], and/or elicitors of defense-related proteins in plants such as salicylic acid [16,36,37,75,102,103].

The recent improvements in *Vitis* cryopreservation are associated with improving the shoot tip quality and uniformity [95,97], pretreatment and preculture conditions, and adding antioxidants and elicitors of defense proteins in shoot tip pretreatment and preculture media [16,35,36,37,76,102].

Marković et al. [97] described a simple and effective step to provide uniform and actively growing shoot tips for grapevine cryopreservation protocols. Single-node micro-cuttings, sourced from in vitro stock cultures, were cultured for two weeks on fresh shooting medium, prior to the excision of apical shoot tips (Figure 1C). The grapevine shoot tips produced from these micro-cuttings had higher regrowth after LN exposure compared to those taken directly from in vitro plantlets. In addition, adding benzylaminopurine (BA) or zeatin riboside (ZR) in the shooting medium had a similar positive impact. Using this protocol, micro-cuttings (1.5 cm in length) of grapevine ‘Portan’ (*V. vinifera* L.) were obtained from 2-month-old in vitro plants and grown on shooting medium consisting of ½ Murashige and Skoog medium (MS) [114], containing 20 g L^−1^ sucrose, 7 g L^−1^ agar, and 1 μmol BA or ZR in Petri dishes at a density of 20 micro-cuttings per plate (Figure 1C). The plates with micro-cuttings were cultured for 2 weeks at 24 ± 2 °C, in a 12 h photoperiod, before uniform apical shoot tips (1 mm length; Figure 1D) were harvested and cryopreserved. In addition to higher regrowth, the shoot tips sampled from micro-cuttings cultured in shooting medium containing BA or ZR produced shoots that were more homogeneous and vigorous compared with the shoot tips sampled directly from in vitro stock plants. Shoot tips positioned at different nodes of a shoot are in varying physiological stages and, as a result, exhibit a range of sizes that differ in their response to cryogenic treatments, affecting cryopreservation results [16,46,97]. The use of micro-cuttings ensures the production of a large number of relatively homogeneous shoot tips, minimizes the physiological effects of apical dominance in the in vitro stock cultures, and increases the chances of a positive and uniform response to subsequent cryogenic treatments [36,97,115].

In recently developed protocols, micro-cuttings have been grown on a pretreatment medium supplemented with salicylic acid (SA) [16], GSH, and AsA, in addition to BA, to reduce the generation of reactive oxygen species (ROS) during cryopreservation procedures [35,36,37,76,102]. Pathirana et al. [16] showed that the pretreatment of donor plantlets with 0.1 mM SA resulted in a higher level of shoot regrowth of the cryopreserved shoot tips of four *V. vinifera* cultivars (Sauvignon blanc, Riesling, Gewurztraminer, and Gruner Veltliner) and two rootstocks—‘Millardet et de Grasset 41B’ (*V. vinifera Chasselas* × *V. berlandieri*) and ‘Schwarzmann’ (*V. riparia × V. rupestris*). In addition, they found that the shoot tips of rootstock ‘Millardet et de Grasset 41B’ could regrow following cryopreservation, albeit at a low percentage (7%), although only when those micro-cuttings were grown on pretreatment media supplemented with SA. The inclusion of 1 mM AsA and 1 mM GSH (reduced form) in combination with 0.2 mg·L^−1^ BA and 0.1 mM SA in the pretreatment medium improved the viability of cryopreserved shoot tips from a wide range of *Vitis* species [36,37,76,102].

For some plant species, such as kiwifruit [108], potato [109,116], and apple [110], the cold-hardening of in vitro stock cultures improved the regrowth and quality of the regenerated plants after cryopreservation. In the studies of Ganino et al. [94] and Benelli et al. [90], the shoot tips of the rootstock ‘Kober 5BB’ (*V. berlandieri* × *V. riparia*) were harvested from in vitro stock shoots that were cold acclimated at 4 °C for 2 and 3 weeks, and cryopreserved using vitrification and encapsulation-vitrification, respectively. Regardless of the cryo-technique, they experienced nil or unsatisfactorily low levels of regrowth after cryoexposure. Bettoni et al. [37] found that the cold-hardening of in vitro shoots at 5 °C for 2 weeks did not significantly improve the regrowth of shoot tips from 12 *Vitis* species after cryopreservation using the droplet-vitrification technique. Nevertheless, cold-hardening may be helpful for the cryoprocessing of some untested *Vitis* species and/or cultivars, as shown by Zhao et al. [89] in *V. vinifera* cv. Cabernet Franc. They showed that the shoot tips of Cabernet Franc could be regenerated following cryopreservation by encapsulation-dehydration, albeit at a low percentage (12.5%), only when the shoot tips were harvested from 3- to 4-month-old in vitro plants that had been cold acclimated for an additional one month at 5 °C.

Shoot tip size affects the success of cryopreservation—typically, shoot tips measuring 1–3 mm in length are most often used for cryopreservation [64,76,95]. In grapevine, 1 mm shoot tips were the most preferred explant and could be effectively cryopreserved, regardless of the cryoprocedures (Table 1; Figure 1D). Comparing the post-LN regrowth levels of four sizes (0.5, 1.0, 1.5 and 2.0 mm) of *Vitis* shoot tips, following an encapsulation-dehydration protocol, Wang et al. [64] found that 1 mm shoot tips produced the highest regrowth level (65%), with larger or smaller shoot tips having lower regrowth levels. Similar results were also reported by Marković et al. [95] and Bettoni et al. [76] using a droplet-vitrification protocol.

Preculturing shoot tips is a necessary step to minimize the cell membrane injury caused by the dehydration processes, and to obtain high shoot regrowth levels in cryopreserved shoot tips [25,27,32]. Shoot tips excised from in vitro stock cultures are cultured on preculture medium with an increased osmotic potential for several hours or days, prior to cryogenic treatments. Sucrose is the most frequently used osmotic agent and has been tested for a wide range of plant genera [46,67]. The sucrose concentration in preculture medium typically ranges from 0.1 to 1.0 M, and can be used at either consistent or increasing concentrations over time [16,31,36,37,81,95,104]. The addition of antioxidants and elicitors of defense proteins to preculture media had a positive impact on the improvement of plant recovery and the quality of plantlets in cryopreserved shoot tips of grapevine [35,36,37,65,117]. Bi et al. [35] reported that grapevine shoot tips precultured in ½ MS medium containing 0.3 M sucrose for 3 days exhibited a low viability level after LN exposure. However, the inclusion of either 0.16 mM GSH or 0.14 mM AsA to the preculture medium produced much higher recovery levels than 0.3 M sucrose alone, with the highest recovery being in cryopreserved shoot tips precultured with a combination of sucrose, GSH, and AsA. Similar improvements in recovery levels, by adding 0.3 M sucrose, 0.1 mM SA, 1 mM AsA, and 1 mM GSH (reduced form) to preculture media during the cryopreservation process, have been reported by Volk et al. [36] and Bettoni et al. [37].

## 4. Methods for Shoot Tip Cryopreservation

Ezawa et al. [84] and Plessis et al. [85,86] pioneered *Vitis* shoot tip cryopreservation, using the classical freezing technique (two-step cooling) to cryopreserve the shoot tips of *V. labrusca* ‘Campbell Early’, ‘Buffalo’ and Delaware, and the *V. vinifera* cultivar Chardonnay. Advances in plant cryobiology in the 1990s, using *Asparagus officinalis* [118], *Citrus* [119], *Solanum* [120], and *Pyrus* [121], simplified effective vitrification [119] and the development of encapsulation/dehydration protocols. In these protocols, shoot tip cells are dehydrated either osmotically or physically prior to plunging them directly into LN, without the need for a programmable freezer. Vitrification and encapsulation methods result in higher freezing rates than the traditional two-step cooling technique [27,82,122]. Vitrification removes freezable water from cells through osmosis, following the precultured shoot tips being exposed to highly concentrated plant vitrification solution (PVS). The vitrification solution consists of a concentrated mixture of penetrating and non-penetrating cryoprotectant substances [123]. PVS2 [119] and PVS3 [124] are the most frequently used PVSs. The former consists of 30% (*w*/*v*) glycerol, 15% (*w*/*v*) ethylene glycol, 15% (*w*/*v*) dimethyl sulfoxide (DMSO), and 0.4 M sucrose in MS liquid medium [119], while the latter comprises 50% (*w*/*v*) sucrose and 50% (*w*/*v*) glycerol [124]. PVS2 has been tested on a wide range of crops [125], including grapevine (Table 1). In encapsulation/dehydration protocols, shoot tips are encapsulated in calcium alginate beads, before air drying in a laminar flow hood or in the presence of silica gel to remove water from the cells. They are usually dehydrated to a moisture content of 15–30% fresh weight basis (FWB) before LN storage [63,88,104].

Since the 1990s, there have been many reports of cryopreservation methods for grapevine, with a wide range of success levels. These methods can be classified into two-step cooling [84,85,86,87,89,91], vitrification [31,32,33,64,92,93,94,98,99,100], encapsulation-dehydration [33,63,64,85,86,88,89,91,95,99,104], encapsulation-vitrification [90], droplet-vitrification [16,35,36,37,51,62,76,95,96,97,101,102,103], and V cryo-plate [76] (Table 1). Some practical and useful resources on *Vitis* cryopreservation and cryotherapy can be found in recent review papers [45,52,67,79,81], thesis documents [65,66,117], practical guides [126], and an eBook training module on *Vitis* shoot tip cryopreservation [127].

### 4.1. Two-Step Cooling

Ezawa et al. [84] were the first to report a two-step cooling protocol for *Vitis* shoot tip cryopreservation, in which the effects of the plant material being collected in different seasons, from field-grown grapevines, and pre-freezing temperatures were tested. The shoot tips (1–2 mm) harvested from field plants were surface sterilized with 70% ethanol for 30 s, followed by 10% sodium hypochlorite solution (1% sodium hypochlorite) and 0.1% Tween^®^ 20 for 15 min. The shoot tips were then treated with cryoprotectant solution containing 10% DMSO and 60 g L^−1^ glucose at 20 °C for 2 h, and progressively cooled at a rate of 0.5 °C min^−1^, from 20 °C to −20, −30, and −40 °C, followed by immersion in LN. The cryopreserved shoot tips were thawed in a water bath at 38 °C and transferred to recovery medium. They found that the shoot tips collected in November and December, from Hokkaido Research Station fields in Japan, and pre-frozen to −30 °C before LN immersion gave better levels of survival compared with those collected in October and pre-frozen to −20 and −40 °C, while the survival percentage of ‘Buffalo’ cryopreserved shoot tips was about 80%, and almost all the ‘Campbell Early’ shoot tips only formed callus [84].

Plessis et al. [85,86] combined encapsulation-dehydration and two-step cooling to cryopreserve shoot tips from in vitro-grown plants of *V. vinifera* cultivar Chardonnay. They harvested axillary shoot tips from 7- to 8-week-old in vitro plants and encapsulated them in alginate beads, then pre-cultured them in sucrose-enriched medium (0.3 to 1.5 M in Plessis et al. [85]; 0.3 to 1 M in Plessis [86]), before conducting partial dehydration by air-drying for 4 h in the air current of a laminar flow hood (bead moisture content of 20% ± 5%, FWB). This was followed by cooling at a rate of 0.5 °C min^−1^, from 20 °C to −80 °C, and then immersion in LN. The cryopreserved beads were slowly rewarmed in air at room temperature for about 15 min. They found that encapsulation and step-wise preculturing, with increasing sucrose concentrations, eliminated the deleterious effects of direct preculturing with high sucrose concentrations [85]. With this protocol, *Vitis* shoot tips were successfully cryopreserved for the first time, with 24–30% recovery reported [85,86]. While this protocol was effective to cryopreserve the shoot tips of *V. vinifera* cultivar Chardonnay, Miaja et al. [87] did not report the same success in three other *V. vinifera* cultivars—Barbera, Nebbiolo, and Brachetto.

Zhao et al. [89] further improved the protocol by harvesting axillary shoot tips from 3- to 4-month-old in vitro plants that had been cold acclimated for one month at 5 °C, followed by encapsulation, partial desiccation, and slow freezing. In this protocol, the cold-acclimated shoot tips were encapsulated in 3% calcium alginate beads and precultured at 5 °C in a medium, with daily increases in the sucrose concentrations from 0.1 to 1 M, desiccated to 21% (FWB), followed by slow cooling to −40 °C at a rate of −0.2 °C min^−1^, before immersion in LN. With these optimized parameters, the regrowth percentages ranged between 15 and 40% for *V. vinifera* cvs. Cabernet Franc, Chardonnay and Fengh-51, and the rootstock LN33 hybrid (*Vitis* L.). Using protocols similar to that described by Zhao et al. [89], Zhai et al. [91] achieved an average of 36% recovery after cryopreservation in four *Vitis* cultivars.

### 4.2. Encapsulation-Dehydration

This procedure is based on the technology developed for producing artificial seeds. As described above, Fabre and Dereuddre [120] were the first to apply the encapsulation-dehydration protocol for cryopreserving *Solanum* shoot tips. Plessis et al. [85,86] were the first to use this method to cryopreserve the shoot tips of *V. vinifera* cv. Chardonnay. As for the two-step cooling method, the shoot tips were encapsulated, stepwise precultured with increasing sucrose concentrations (0.3 to 1 M), and dehydrated by air-drying in a laminar flow hood for 4 h (bead moisture content of 20% ± 5%, FWB), prior to direct immersion in LN. The cryopreserved shoot tips were slowly rewarmed in air at room temperature for about 15 min, and then plated onto growth medium. This procedure resulted in 30% shoot tip survival, but the regrowth levels were not specified [86].

Wang et al. [88] further improved the encapsulation-dehydration protocol by optimizing the water content of the encapsulated shoot tips, rewarming methods, and post-culture medium of the cryopreserved shoot tips. They excised shoot tips (1 mm) from 4-week-old in vitro cultures and encapsulated them using 3% sodium alginate solution made of ½ MS liquid medium, and 2 M glycerol + 0.4 M sucrose in 0.1 M CaCl_2_ solution composed of 2 M glycerol + 0.4 M sucrose in liquid MS medium for 30 min at room temperature. The encapsulated beads (4 mm diameter), each containing one shoot tip, were then stepwise precultured with increasing sucrose concentrations of 0.25, 0.5, 0.75 and 1 M, each for one day. The precultured beads were partially dehydrated in a laminar flow hood for 6 and 7.5 h to achieve 17.6 and 15.6% bead moisture contents for *V. vinifera* ‘Superior’ and the LN33 hybrid (*Vitis* L.), respectively. The beads were then transferred to cryovials and directly immersed in LN. The frozen cryovials were rapidly thawed in a water bath at 40 °C for 3 min, and then the beads were cultured in ½ MS medium containing 1 mg L^−1^ BA and 0.1 naphthaleneacetic acid (NAA) for 2 days in the dark, and then transferred to light conditions. With this optimized protocol, 60 and 40% shoot tip survival levels were obtained for the LN33 hybrid and cultivar ‘Superior’ after LN exposure, respectively. Preculturing the beads is the primary step that induces desiccation tolerance in the encapsulation-dehydration protocols, and the progressive increase in sucrose concentration avoids the deleterious effects of direct high sucrose exposure [80,85,88,128]. Fast thawing at 40 °C, using the encapsulation-dehydration protocol, improved the survival of the cryopreserved shoot tips for both the LN33 hybrid and cultivar ‘Superior’, compared to slow warming at room temperature for 15 min, which was described by Plessis et al. [85,86].

The success of the encapsulation-dehydration protocol depends upon the extent of dehydration and the moisture content of the encapsulated beads of the plant material before freezing [45,52,67,88]. The dehydration period can vary depending on the ambient temperature, humidity, and air flow velocity, especially when air-drying in a laminar flow hood. Wang et al. [88] compared dehydration using air-drying in a laminar flow hood and silica gel, finding that the survival levels of grapevine rootstock LN33 were not dependent on the dehydration method, but, instead, on the water content of the beads. Although the two dehydration methods produced similar recovery results, the air-drying method was difficult to replicate, due to environmental variations; therefore, dehydration using silica gel was preferred [27,45].

Plessis et al. [85,86] reported that the highest survival (30%) of cryopreserved shoot tips occurred when the beads encapsulating the shoot tips were partially dehydrated for 4 h in air, to reach a moisture content of 20%. Similarly, Zhao et al. [89] showed that the encapsulated shoot tips of Cabernet Franc could be regenerated (12.5%) from alginate beads desiccated to a moisture content of 21.8%. In the study of Bayati et al. [63], 12 h of dehydration in air (bead moisture content not specified) resulted in 59% shoot tip recovery in the cryopreserved beads of *V. vinifera* ‘Black’. For *V. vinifera*, cv. Portan, 37% regrowth was reported when the encapsulated shoot tips were dehydrated for 4 h on silica gel, to a moisture content of 22.3% [95]. Recently, AlMousa and Hassan [104] found that the highest survival (33%; regrowth not specified) was achieved when precultured beads were dehydrated by air-drying in a laminar flow hood for 6 h, to a moisture content of 18.4%. These results indicate that the optimal water content for the recovery of encapsulated shoots is not only species specific, but also cultivar specific. Thus, in order to achieve higher regrowth levels after LN exposure, it is necessary to test the effect of dehydration on recovery, to identify the minimum water level that the shoot tips can survive in, even without LN exposure [52,88].

Encapsulation-dehydration methods overcome some of the problems associated with the sensitivity of some plant material to vitrification solutions, because sucrose is the only osmotic agent that induces desiccation [77,82,129]. However, for most of the grapevine studies listed in Table 1, vitrification methods produced higher levels of shoot tip recovery than encapsulation-dehydration methods.

### 4.3. Vitrification

Vitrification-based cryopreservation is the most widely applied method for cryopreserving plant shoot tips [68,69]. The key to success when using this method is to induce shoot tip dehydration tolerance with highly concentrated vitrification solutions, to avoid ice crystal formation during freezing and warming [68,80,130]. The optimal exposure duration and temperature of vitrification solutions need to be determined to produce a high level of shoot recovery after vitrification. The optimal exposure duration depends upon the plant species, explant conditions, cryoprotectant exposure temperature, as well as preculture/pretreatment conditions [46,83,130,131,132].

For vitrification, pre-cultured shoot tips are treated with a loading solution (LS) composed of 2 M glycerol + 0.4 M sucrose [133], and then exposed to PVS. The loading treatment alleviates the osmotic stress or chemical toxicity imposed by the direct exposure to PVS [133]. The explants are transferred to cryovials containing small volumes (1–1.5 mL) of fresh vitrification solution, and plunged into LN. The cryovials are then rapidly thawed in a water bath at 40 °C, and the shoot tips are placed into unloading solution (ULS; 1.2 M sucrose) to remove the cryoprotectants before being cultured in recovery medium [31].

**Figure 2:**
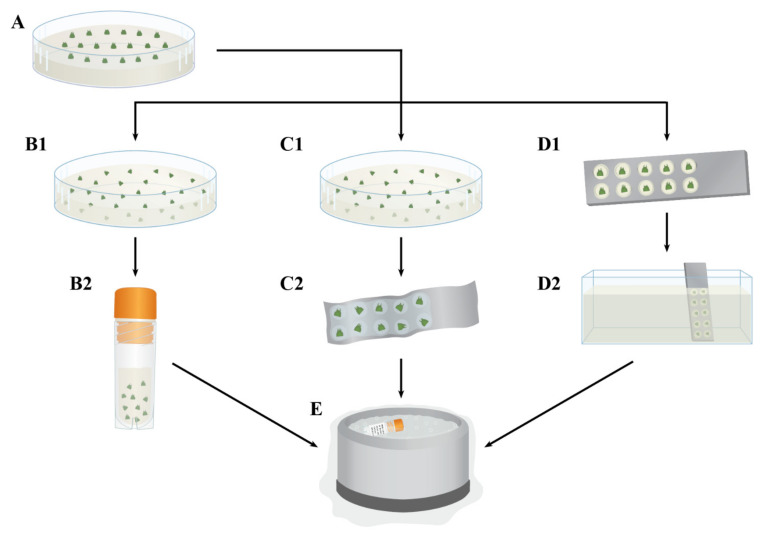
Major steps of grapevine shoot tip cryopreservation by vitrification (**B1**,**B2**; [31,32]), droplet-vitrification (**C1**,**C2**; [35,36,37]) and V cryo-plate (**D1**,**D2**; [76]). Shoot tips incubated in the preculture medium (**A**), osmoprotected in loading solution (**B1**,**C1**) or first attached to cryo-plates using calcium alginate solution (**D1**) after which cryo-plates with shoot tips attached are osmoprotected in loading solution (**D2**). Osmoprotected shoot tips either attached to cryo-plate (V cryo-plate) or not (vitrification and droplet-vitrification) are dehydrated in PVS2 (**D2**; V cryo-plate). Shoot tips are transferred to cryovials containing a small volume (1–1.5 mL) of fresh vitrification solution (**B2**; vitrification) or a thin layer of PVS2 placed on sterile aluminum foil strips (**C2**; droplet-vitrification) or cryo-plates followed by liquid nitrogen exposure (**E**).

Matsumoto et al. [31] were the first to report a vitrification cryopreservation method for four cultivars of in vitro-cultured grape shoot tips, and this protocol was further extended to 10 *Vitis* accessions (Figure 2(B1,B2)) [32]. In this latter study [32], they excised axillary shoot tips (1 mm long) from 4- and 5-month-old in vitro stock plants and pre-cultured them on solidified ½ MS containing 0.3 M sucrose for 3 days at 25 °C (Figure 2A), then osmoprotected them with loading solution (2 M glycerol + 0.4 M sucrose in MS medium) for 20 min at 25 °C (Figure 2(B1,B2)). The shoot tips were subsequently dehydrated with a 50% (half-strength) PVS2 for 30 min at 0 °C, followed by full-strength PVS2 for 50 min at 0 °C. The shoot tips were then placed in cryovials containing 1 mL of fresh PVS2 and directly plunged into LN (Figure 2B). The cryovials were rapidly warmed in water at 40 °C for 1 min, and PVS2 was drained and replaced with ULS (1.2 M glycerol in MS medium), followed by incubation for 20 min. The shoot tips were then transferred onto sterilized filter paper discs over a recovery medium (½ MS medium containing 1 mg L^−1^ BA, 3% sucrose, and 0.2% gellan gum) for one day, before being transferred to fresh paper discs. This modified vitrification protocol (two-step vitrification) gave an average of 64% post-cryopreservation recovery [32], which was a much higher recovery than the conventional one-step vitrification method [31,32]. By preculturing shoot tips with increasing sucrose concentrations of 0.25, 0.5, and 0.75 M for 3 days, each for one day, and fast thawing, Wang et al. [33] obtained about 40% shoot regrowth for the LN rootstock and scion ‘Superior’.

Using a classic one-step vitrification protocol, Fabbri et al. [92] and Ganino et al. [94] found that, for the rootstock ‘Kober 5BB’ (*V. berlandieri* × *V. riparia*), the shoot tips excised from in vitro stock shoots, either with or without cold acclimation, had low survival using a vitrification cryoprocedure. Unlike *V. vinifera* [31], they reported no shoot tip regrowth post-cryopreservation, even though the cryopreserved shoot tips showed signs of survival [92,94].

Shoot tips from *V. vinifera* ‘Salty Kodari’ were also cryopreserved using alternative loading and vitrification solutions, followed by one-step vitrification [93]. Shatnawi et al. [93] proposed an alternative to traditional LS (2 M glycerol + 0.4 M sucrose) and PVS2, in which pre-cultured shoot tips (MS + 0.3 M sucrose for 1 day) were treated with MS containing 5% (*w*/*v*) DMSO, 5% (*w*/*v*) glycerol, and 5% (*w*/*v*) sucrose for 20 min at 0 °C, followed by either PVS2 or MPVS2 (15% (*w*/*v*) glycerol, 20% (*w*/*v*) DMSO, 15% ethylene glycol + 14% (*w*/*v*) sucrose in MS liquid medium) exposure, before immersion in LN. Between 47% and 55% shoot regrowth was achieved in shoot tips treated with PVS2 or MPVS2 for 40 min at 0 °C, respectively. Whilst this protocol was effective for cryopreserving one accession of *V. vinifera*, Dal Bosco et al. [99] did not experience the same success with another 12 *Vitis* accessions, including seven *V. vinifera* cultivars, after LN exposure. Additional studies using these alternative solutions are required to validate this protocol.

### 4.4. Encapsulation-Vitrification

The encapsulation-vitrification method combines encapsulation-dehydration and vitrification procedures; encapsulated shoot tips are osmoprotected by LS and exposed to PVS2, prior to direct immersion in LN [27,52,130,134]. This method was designed by Matsumoto and his colleagues to process a large number of explants, combining the advantages of the easy manipulation of encapsulated explants and fast dehydration by vitrification [125,128,130,135,136,137]. This method is less well studied than other cryoprocedures for grapevine (Table 1). Benelli et al. [90] investigated the efficiency of the encapsulation-vitrification method on cryopreserve shoot tips from rootstock ‘Kober 5BB’, which was previously shown, by Ganino et al. [94], to be difficult to cryopreserve by vitrification. Benelli et al. [90] excised apical and axillary shoot tips (1–2 mm) from in vitro cultures that had been cold acclimated for 3 weeks at 4 °C and then encapsulated in 3% calcium alginate beads. The beads were transferred to cryovials and incubated in PVS2 at 0 °C for 30 or 90 min before immersion in LN. The cryovials that had been immersed in LN were rapidly thawed in a water bath at 40 °C, and then beads were cultured in recovery medium. Shoot tips of rootstock ‘Kober 5BB’ could be regenerated following encapsulation-vitrification cryopreservation, albeit at unsatisfactorily low percentages (regrowth level not specified by the authors).

### 4.5. Droplet-Vitrification

Droplet vitrification is derived from the DMSO droplet methods developed by Kartha et al. [138] and Schäfer-Menuhr et al. [139,140,141] for freezing cassava and potato shoot tips, respectively. Initially, Kartha et al. [138] cryopreserved cassava shoot tips in droplets of cryoprotectant solution (15% DMSO + 3% sucrose, 15 min at room temperature) on aluminum foil strips using slow cooling. Schäfer-Menuhr et al. [139,140,141] further modified this protocol by cryoprotecting potato shoot tips in 10% DMSO, for 2 h at room temperature, and placing them onto a droplet of cryoprotectant solution on foil strips, before plunging them directly into LN. The methods proposed by Kartha et al. [138] and Schäfer-Menuhr et al. [139,140,141] have not been widely used. Panis et al. [142] reported a droplet-vitrification protocol for the cryopreservation of banana shoot tips, adding the LS step and PVS2 as a vitrificant agent. This method is applicable to a wide range of genotypes and appears to have overcome the genotype specificity problem, which was a bottleneck for the wider application of plant cryopreservation. Droplet-vitrification has been successfully applied to many important plant species and is widely used in genebanks for cryopreserving vegetatively propagated crop collections [22,68,135,143].

Droplet-vitrification makes use of ultra-fast shoot tip cooling and warming rates, an important requirement for successful cryopreservation protocols based on vitrification [142]. High cooling rates are achieved by placing shoot tips onto droplets (1–5 μL per shoot tip) of cryoprotective solution on aluminum foil, with direct exposure to LN, compared to traditional vitrification methods, where shoot tips are cryopreserved within capped vials, with the cryoprotective medium (0.5–1.5 mL) in the vial [46,130,142,143,144,145,146].

The droplet-vitrification method is frequently used for grapevine germplasm and has been demonstrated to be the most effective cryopreservation method across diverse *Vitis* species (Table 1). Marković et al. [95,97] were the first to successfully apply droplet-vitrification to the shoot tip cryopreservation of grapevine ‘Portan’ (*V. vinifera* L.). Briefly, in their study, micro-cuttings grown for 2 weeks on shooting medium (½ MS containing 20 g L^−1^ sucrose, 7 g L^−1^ agar, and 1 μmol BA or ZR) served as source material for uniform apical shoot tips (1 mm) for cryopreservation. The shoot tips were precultured in MS supplemented with sucrose, either fixed (0.1 M for 24 h [95]) or increased in increments (every 12 h of 0.25 M, 0.5 M, 0.75 M, and 1 M [97]), and then treated with LS (2 M glycerol + 0.4 M sucrose in MS medium) for 20 min at room temperature. Osmoprotected shoot tips were exposed either directly to full-strength PVS2 for 50 min at 0 °C [95], or half-strength PVS2 for 30 min at room temperature and then full-strength PVS2 for 50 min at 0 °C [97]. PVS2-treated shoot tips were transferred to 5 μL of PVS2 droplets, placed on sterile aluminum foil strips, and directly immersed in LN. For warming, the aluminum foils containing the shoot tips were immersed, for 20 min, in ULS (1.2 M sucrose in MS medium) at room temperature, and transferred to recovery medium. Similar regrowth levels were observed in the cryopreserved shoot tip of grapevine cultivar ‘Portan’ exposed either directly to full-strength PVS2 (50%), or half-strength PVS2 and then full-strength PVS2 (44%) (two-step vitrification) prior to LN exposure.

Marković et al. [34] subsequently applied the optimized droplet-vitrification protocol [95], using the two-step vitrification method, to nine *V. vinifera* accessions, and reported that shoot tip regrowth ranged from 0 to 70%, indicating a genotype-specific response to the protocol tested. Grapevines Merlot (70%), ‘Portan‘ (50%), Cabernet Sauvignon (46.6%), and Chardonnay (30%) had acceptable levels of regrowth compared to Maraština (11%) and four *V. vinifera* cultivars, Pošip, Škrlet, Pinot noir, and Plavac mali, that did not exhibit any shoot tip regrowth after LN.

Pathirana et al. [16,62] further optimized the droplet-vitrification protocol by excising shoot tips from micro-cuttings grown in shooting medium (pretreatment) supplemented with 0.1 mM SA, followed by serial dehydration in sucrose-enriched medium of 0.25, 0.5, 0.75 and 1 M (each for 24 h), and adding a post-thaw culture medium enriched with sucrose (one day per step). Pre-cultured shoot tips (1–1.5 mm) were treated in LS (2 M glycerol + 0.4 M sucrose in MS medium; Figure 2(C1)) and then were immersed in PVS2 (one-step vitrification) for 36–43 min at 0 °C. PVS2-treated shoot tips were placed onto droplets of PVS2 on foil strips (Figure 2(C2)) and plunged into LN (Figure 2E). For thawing, the aluminum foil strips containing the shoot tips were placed in ULS (1.2 M sucrose in MS medium) at room temperature for 20 min and incubated in post-thaw culture medium enriched with 0.6 M sucrose for 24 h, before being transferred to recovery medium in darkness for 1 week, followed by a transfer to light conditions. This cryoprotocol resulted in 7–45% shoot regrowth for 10 *Vitis* accessions [16,62]. They found a strong genotype effect with 6 of the 10 accessions tested, which had regrowth levels below 30%, with rootstock ‘Millardet et de Grasset 41B’ showing the least regrowth potential (7%).

Recent reports suggest that droplet-vitrification procedures show promise in overcoming genotype-specific responses in *Vitis* species, allowing the first efforts for the implementation of cryopreserved *Vitis* genebank collections to be initiated [35,36,37].

Bi et al. [35,51] described a successful droplet-vitrification protocol that was applied to eight *Vitis* accessions (six *V. vinifera* genotypes and two *V. pseudoreticulata* genotypes). The procedure involved the excision of shoot tips from micro-cuttings, incubation for 3 days in preculture medium containing 0.3 M sucrose, 0.16 mM GSH, and 0.14 mM AsA, treatment of the pre-cultured shoot tips for 20 min at room temperature with LS (2 M glycerol + 0.4 M sucrose in MS medium), and exposure to two-step vitrification on PVS2 (half-strength PVS2 for 30 min, followed by full-strength PVS2 for 50 min at 0 °C). Then, the PVS2-treated shoot tips were transferred into 2.5 μL PVS2 carried on aluminum foil, prior to direct immersion in LN. The cryopreserved shoot tips were thawed in ULS (1.2 M sucrose in MS medium) at 24 °C for 20 min, and post-cultured on ½ MS supplemented with 0.6 M sucrose and 7 g L^−1^ agar for 1 d in the dark, before being transferred to recovery medium in light conditions. With this method, the shoot tip regrowth levels ranged from 24 to 72% and averaged at 50.5% across the eight *Vitis* accessions.

More recently, Volk et al. [36] outlined a droplet-vitrification protocol for *Vitis* species through improved shoot tip quality, pretreatment and preculture conditions, vitrification exposure duration, and regrowth medium, to achieve consistent regrowth after cryopreservation. This protocol combines multiple pretreatments, many of which have been previously demonstrated to be effective in *Vitis*. In this protocol, shoot tips (1–1.5 mm) were excised from micro-cuttings grown on pretreatment medium containing 0.2 mg L^1^ BA, 1 mM SA, 1 mM GSH (reduced form), and 1 mM AsA for 2–3 weeks. The shoot tips were then pre-cultured on ½ MS medium containing 0.3 M sucrose, 0.1 mM SA, 1 mM AsA, and 1 mM GSH (reduced form) for 3 days at 25 °C in the dark. The precultured shoot tips were treated with LS (2 M glycerol + 0.4 M sucrose in MS medium) for 20 min at 22 °C, followed by half-strength PVS2 for 30 min at 22 °C and full-strength PVS2 for 90 min at 0 °C. The PVS2-treated shoot tips were placed onto a thin layer of PVS2 on foil strips and plunged into LN. The cryopreserved shoot tips were warmed in ULS (1.2 M sucrose in MS medium) for 20 min at 22 °C, and placed in recovery medium #1 (½ MS macroelements without ammonium, full-strength MS microelements, and *Vitis* vitamins (100 mg·L^−1^ myo inositol, 10 mg·L^−1^ thiamine HCl, 1 mg·L^−1^ nicotinic acid, 1 mg·L^−1^ pyridoxine HCl, 1 mg·L^−1^ Ca pantothenate, 0.01 mg·L^−1^ biotin, and 2 mg·L^−1^ glycine) supplemented with 0.6 M sucrose and 8 g·L^−1^ agar) overnight in the dark, and then transferred to recovery medium #2 (½ MS macroelements without ammonium, full-strength MS microelements, and *Vitis* vitamins supplemented with 30 g·L^−1^ sucrose, 0.2 mg·L^−1^ BA, and 8 g·L^−1^ agar) and cultured for 2 weeks at 25 °C in darkness. The shoot tips were then transferred to recovery medium #3 (½ MS macroelements, full-strength MS microelements, and *Vitis* vitamins supplemented with 30 g·L^−1^ sucrose, 0.2 mg·L^−1^ BA, and 8 g·L^−1^ agar) and grown in the light at 25 °C. The improvements in regrowth were achieved by eliminating ammonium from the recovery medium (#1 and #2) during the first 15 days of post-thaw incubation, minimizing the BA concentration in the recovery medium, increasing the agar concentration, and using a plastic sealing film (PVC) that increased the air exchange of cultures. With this optimized protocol, the shoot tip regrowth levels ranged from 24 to 43% and averaged at 35% across nine *Vitis* species.

Bettoni et al. [37] successfully repeated a similar protocol, proposed by Volk et al. [36], in 13 genotypes, representing 12 *Vitis* species (Table 1). The regrowth levels of least 43% and the successful replication by two technicians suggest that this method is ready for implementation. The same team demonstrated that *Vitis* shoot tips might be cryopreserved without introducing the accession into the tissue culture first [102,103]. In their study, nodal sections were harvested from plants grown in a growth chamber, surface sterilized (70% isopropanol for 1 min, followed by 5% sodium hypochlorite and 0.1% Tween 20 for 5 min) and plated on pretreatment medium for 2 weeks, after which uniform shoot tips (1 mm) were dissected and pre-cultured for 3 days. The pretreatment and preculture media were those described above by Volk et al. [36], with addition of the 1.5% (*v*/*v*) plant preservation mixture (PPM^®^) to reduce microbial contamination. The precultured shoot tips were treated with LS for 20 min, followed by two-step vitrification on PVS2 (half-strength PVS2 for 30 min at 22 °C, followed by full-strength PVS2 for 30–40 min at 0 °C) prior to immersion in LN. This method resulted in regrowth levels of 43–64% for two *V. vinifera* cvs. Chardonnay and Riesling, and rootstock ‘Oppenheim’ (SO4; *V. berlandieri* × *V. riparia*) [102]. The rootstock ‘Oppenheim’ has a similar genetic background to ‘Kober 5BB’ (*V. berlandieri* × *V. riparia*), which was previously shown, by Fabbri et al. [92] and Ganino et al. [94], to be recalcitrant to cryopreservation using a one-step vitrification protocol. Using the protocol proposed by Bettoni et al. [102], ‘Oppenheim’ had a regrowth percentage of 48% after cryoexposure. The use of explants directly from the greenhouse, or possibly even from field-grown plants, could further increase the efficiency of cryopreserving cultivars in genebank collections; this needs to be explored for additional *Vitis* accessions.

### 4.6. V Cryo-Plate

A vitrification protocol using the aluminum cryo-plate method (V cryo-plate) was recently developed by Yamamoto et al. [147], using ‘Dalmatian’ chrysanthemum (*Tanacetum cinerariifolium*) in vitro shoot tips. The V cryo-plate method uses calcium alginate to attach shoot tips to cryo-plates. The shoot tips are then osmoprotected, PVS2-dehydrated, and immersed directly in LN [148]. Cryo-plate methods simplify the handling of shoot tips at different stages of cryopreservation [46]. The shoot tips remain attached to the cryo-plates throughout the whole procedure; therefore, they are easily transferred between solutions, allowing precise control of the treatment exposure duration, and reducing the risk of mechanical injury through handling during the course of the cryopreservation protocol. Fast cooling and warming rates have been reported to result in high regrowth levels after cryopreservation for a diverse range of genetic resources [76,147,148,149,150,151].

Bettoni et al. [76] were the first to report a V cryo-plate protocol for *Vitis* shoot tip cryopreservation. This protocol incorporates optimized pretreatment and preculture media, as well as the shoot tip recovery process using a droplet-vitrification cryopreservation method previously reported [36,37]. In this protocol, micro-cuttings were grown on pretreatment medium for 2 weeks, and then shoot tips (1 mm) were excised and placed on preculture medium for 3 days at 25 °C in the dark. Droplets (~2 μL) of 2% sodium alginate solution, in a calcium-free MS basal medium supplemented with 0.4 M sucrose, were placed into each well in the cryo-plates (No. 2; 37 × 7 × 0.5 mm). Precultured shoot tips (1 mm) were placed individually into each well, and 1.5 μL of sodium alginate solution was added to cover the shoot tips completely. Then, calcium chloride solution (0.1 M calcium chloride in MS basal medium supplemented with 0.4 M sucrose) was added dropwise to the cryo-plate, until all the wells were covered, and kept at 22 °C for 20 min for polymerization. Cryo-plates with shoot tips (Figure 2(D1)) were placed in LS (2 M glycerol + 0.4 M sucrose in MS medium) for 30 min at 22 °C, followed by one-step vitrification on PVS2 for 30–40 min at 22 °C (Figure 2(D2)), prior to immersion in LN (Figure 2E). Cryo-plates with shoot tips were warmed in ULS (1.2 M sucrose in MS medium) at 22 °C for 20 min, and alginate beads were then detached from the cryo-plates and transferred into the recovery medium. This protocol resulted in 68–70% shoot tip regrowth in the *Vitis* accessions *V. aestivalis* and *V. jacquemontii*. Given that this protocol can be easily executed, and high-quality plants were obtained from cryo-exposed shoot tips, it seems to be a practical and promising *Vitis* cryopreservation methodology. However, further work is required to determine the applicability of this protocol to a wider range of *Vitis* accessions.

## 5. Shoot Tip Cryotherapy

Shoot tip cryotherapy refers to freezing the infected shoot tips in LN to eradicate pathogens [52,55,152]. In 1997, Brison et al. [54] reported the first successful eradication of *Plum pox virus* (PPV) from the infected shoot tips of a *Prunus* rootstock, using a vitrification method. Since then, shoot tip cryotherapy has been successfully applied to eradicate 37 viruses [52,153,154,155,156,157,158,159,160,161,162], two viroids [163], two phytoplasmas [164,165], and one bacterium [166] in 31 plant species. To date, five phloem-limited grape viruses have been eradicated by shoot tip cryotherapy, including *Grapevine fanleaf virus* (GFLV), *Grape virus A* (GVA), *Grapevine leafroll-associated virus-1,-2,-3* (GLRaV-1,2,3) [34,51,62,63,64,167] (Table 2).

### 5.1. Methods for Shoot Tip Cryotherapy

Wang et al. [64] were the first to report the eradication of GVA from *V. vinifera* ‘Bruti’ using vitrification and encapsulation-dehydration cryotherapy. Vitrification and encapsulation-dehydration methods both resulted in 97% virus elimination, analyzed by Western blotting. Applying encapsulation-dehydration cryotherapy, Bayati et al. [63] obtained 42.3% GVA-free plants of *V. vinifera* ‘Black’, analyzed by reverse transcription polymerase chain reaction (RT-PCR). Gribaudo et al. [167] applied encapsulation-vitrification to eradicate GVA and GLRaV-3, and obtained 100% virus-free plants in *V. vinifera* Nebbiolo, analyzed by multiplex RT-PCR.

Pathirana et al. [62] used droplet-vitrification cryotherapy to eradicate GLRa-V-3, -2, and -1 from several *V. vinifera* cultivars, obtaining 100% GLRaV-3-free plants in *V. vinifera* Chardonnay and ‘Lakemont Seedless’, 100% GLRaV-2-free plants in Pinot gris and ‘Sauvignon blanc 316’, and 100% GLRaV-1-free and GLRaV-3-free plants in Sauvignon blanc, with an average shoot regrowth level of 17.7% across the cultivars. The virus eradication frequency was confirmed by double-antibody sandwich enzyme-linked immunosorbent assay (DAS-ELISA). Marković et al. [34] reported the successful eradication of GFLV from *V. vinifera* Chardonnay, and GLRaV-3 from *V. vinifera* Cabernet Sauvignon using droplet-vitrification, obtaining 100% and 77.8% virus-free frequencies, respectively, and a 57% average regrowth level for the two cultivars [34]. Recently, Bi et al. [51] reported that all plants regenerated from cryopreservation were free of GLRaV-3, as confirmed by RT-PCR and microtissue direct RT-PCR (MD RT-PCR), in *V. vinifera* Cabernet Sauvignon and Chardonnay, *V. vinifera* × *V. labrusca* ‘Kyoho’, *V. pseudoreticulata* ‘Hunan-1’, by droplet-vitrification. This report supported the critical role of LN exposure for successful cryotherapy for virus eradication [51].

Testing the effects of various steps of encapsulation-dehydration cryotherapy on virus eradication, Wang et al. [64] found that only the regenerants that recovered after LN exposure produced virus-free plants; the steps before and after freezing in LN failed to eradicate the virus. Virus eradication by freezing in LN, but not by other steps, during cryopreservation procedures was confirmed in droplet-vitrification [51,62] and encapsulation-dehydration [63]. Interestingly, Marković et al. [34] found that 100% and 82.4% of the plants of V. vinifera Cabernet Sauvignon and Chardonnay that recovered from PVS2 treatments without freezing in LN were free of GLRaV-3 and GFLV, respectively, which is very similar to the 100% and 77.8% virus-free plants recovered after cryopreservation. These results were in contrast with the former reports [62,63,64]. More studies are needed to verify if PVS treatments can eradicate viruses on other grape–virus combinations.

### 5.2. Mechanism Involved in Shoot Tip Cryotherapy for Eradication of Phloem-Limited Grapevine Viruses

**Figure 3:**
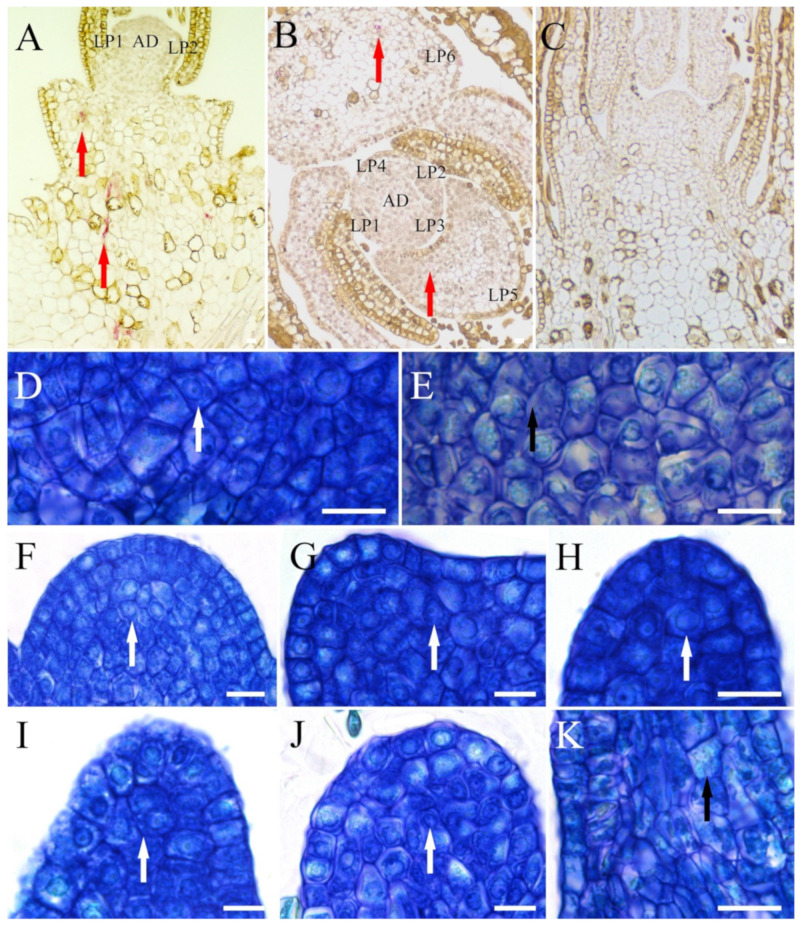
Immunohistological localization of *Grapevine leafroll-associated virus-3* (GLRaV-3) in longitudinal section and cross section of shoot tips and histological observations of cell survival patterns in longitudinal sections of shoot tips following cryotherapy in grapevine cultivar Cabernet Sauvignon. Virus-infected cells are indicated by purple color (red arrows) reactions in (**A**,**B**), whereas no purple color reactions were spot in virus-free cells in (**C**). Surviving cells are densely stained with intact nucleus and nucleolus enclosed in a well-preserved cytoplasm (white arrows) in (**D**) as a positive control, dead cells are cytoplasm condensed with fractured plasma membrane (black arrows) in (**E**) as a negative control. Surviving cells in AD (**F**), and LP 1 (**G**), LP 2 (**H**), LP 3 (**I**), LP 4 (**J**) and dead cells in LP 5 (**K**) following cryotherapy are indicated by white arrows and black arrows. Bars = 10 μm. Source: Bi et al. [51].

The virus is unevenly distributed in the virus-infected shoot tips [55,59,160,161,168]. Immunohistochemical (IHC) observations found that phloem-limited GLRaV-3 was not present in the top-layer cells of the apical dome (AD) and the youngest four leaf primordia (LPs), but was present in the lower parts of the AD, LP 5, and older tissues of the virus-infected shoot tips of V. vinifera Cabernet Sauvignon, leaving about 0.5 mm of the meristematic area free of virus infection [51]. After freezing in LN, living cells were found in the top tissue of the AD and LPs 1–4 of the shoot tips (Figure 3). Few living cells were found in LP 5, and other cells were killed. Thus, plants that recover from cryotherapy can be free of viruses. The results of virus localization and cell surviving pattern provided explanations as to why shoot tip cryotherapy can eradicate phloem-limited grapevine viruses, such as GVA, GFLV, GLRa-V-3, -2, and -1.

### 5.3. Comparison of Virus Eradication Efficiency between the Traditional Methods and Shoot Tip Cryotherapy

Shoot tip culture (also called meristem culture) has traditionally been used to produce virus-free plants [93,169,170,171,172,173,174]. In shoot tip culture, the size of the shoot tips is positively related to survival and shoot regeneration, but negatively proportional to the virus eradication frequency. Therefore, typically, shoot tips of 0.1 to 0.5 mm, containing an AD and 2–4 LPs, are used for virus eradication [64,93,173,174]. The excision of such small shoot tips is difficult and requires skilled lab workers, in comparison to the larger shoot tips (1.0 mm) used in shoot tip cryotherapy (Table 2). It can also be difficult to regenerate plants from tiny shoot tips using traditional shoot tip culture methods [29,52,64,175]. Wang et al. [64] demonstrated that 0.5 to 2.0 mm shoot tips had similar high GVA-free frequencies (97%) by using encapsulation-dehydration cryotherapy, with a 50% to 65% regrowth rate, while 0.2 to 0.4 mm shoot tips could only produce a maximum of 12% GVA-free frequencies by shoot tip culture, with a 75% to 100% regrowth rate. Although shoot tip cryotherapy produced lower shoot regrowth than meristem culture, shoot tip cryotherapy produced much higher virus eradication frequencies than the traditional methods. A shoot tip culture with 0.2 to 0.5 mm shoot tips has been used to obtain plants of rootstock Kalecik Karasi that were 100% free of GLRaV-3 [174], while, in cryotherapy, shoot tips of bigger size (1.0 to 2.0 mm) were used to fully eradicate GLRaV-3 from grapevine Cabernet Sauvignon, Chardonnay, Nebbiolo, ‘Kyoho’, and rootstock ‘Hunan-1’ [34,51,167]. Those studies demonstrated that larger shoot tips could be used in shoot tip cryotherapy for efficient virus eradication, compared to the traditional methods.

Shoot tip culture is more effective in eliminating viruses when it is combined with thermotherapy [175,176], and allows the use of larger shoot tips than those used for shoot tip culture without thermotherapy. Thermotherapy treatment associated with shoot tip culture resulted in virus-free frequencies of 91% for GLRaV-1 and 68% for grapevine *Rupestris stem pitting-associated virus* (GRSPaV) in *V. vinifera* cv. Agiorgitiko [176], and 100% eradication for GFLV, 70% for GVA, 25% for GLRaV-1, 25% for GLRaV-3, and 0% for *Grapevine fleck virus* (GFKV) in rootstock ‘Kober 5BB’ [177]. However, thermotherapy requires a temperature-controlled growth chamber, which is expensive. In addition, thermotherapy is time consuming compared with shoot tip culture.

In shoot tip cryotherapy, the success in eradicating viruses is independent of the size of the shoot tips used [64,160]. Wang et al. [64] tested the effect of shoot tip size (0.5 to 2.0 mm) on survival and GVA eradication frequency after cryotherapy. Their study demonstrated that the size of the shoot tips affected the survival after cryopreservation. Shoot tips that were 1.0 mm had the highest survival, at 65%, while greater or smaller sizes of shoot tips resulted in lower survival. However, the virus eradication frequency was not affected by the size of shoot tips used after cryopreservation, and was at a high level of 97% free of GVA. In the experiments of Bi et al. [51], three sizes of apical shoot tips were used to test regeneration and virus eradication efficiency by droplet-vitrification. Significantly higher survival (75%) and regrowth (59%) levels were obtained in the 1.0 mm shoot tip after cryopreservation. All the plants that recovered from LN were 100% free from GLRaV-3, regardless of the size of shoot tips used. These reports are consistent with the findings obtained in virus eradication by cryotherapy so far [55,59,64,160], confirming, again, that pathogen-free frequencies are independent of the size of shoot tips used for cryotherapy.

Relative to shoot tip culture or thermotherapy followed by shoot tip culture, shoot tip cryotherapy has the following advantages: cryotherapy yields pathogen-free plants at a high frequency; avoids the time-consuming excision of very small tissues, which requires skillful technicians; does require special equipment in addition to that typically available in a plant tissue culture laboratory. In summary, cryotherapy facilitates the treatment of a large number of samples quickly and simultaneously, reducing costs and making procedures shorter compared to thermotherapy followed by shoot tip culture [52,54,64,81,152,153,154,155,175,178].

## 6. Conclusions and Future Prospects

The availability of and easy access to *Vitis* genetic resources are essential for future breeding program advances. Shoot tip cryopreservation is a valuable technique for the safe, long-term conservation of *Vitis* genetic resources that complements traditional field and in vitro germplasm collection activity.

Reliable cryopreservation methods that result in high levels of regrowth are crucial to develop and implement backup collections in LN. Therefore, it is critical to ensure that the *Vitis* accessions being cryopreserved successfully recover normal plants. To avoid the production of somaclonal variants, it is important to recover shoot tips directly, without a callus intermediate [45].

Shoot tip cryopreservation protocols depend on tissue culture to regenerate healthy plants. Therefore, efficient cryopreservation protocols should include optimized micropropagation systems. Culture media formulation and growth conditions must be established to induce favorable physiological conditions for shoot tip donor plants, as well as appropriate conditions for shoot tip recovery post cryopreservation.

The success of cryopreservation protocols depends on a combination of numerous factors, such as shoot tip quality, pretreatment and preculture conditions, osmoprotection and vitrification dehydration steps, as well as the recovery process after cryoexposure, and these aspects can be genotype as well as species specific. One of the main factors to ensure success in *Vitis* cryopreservation is to obtain uniform, actively growing shoot tips. This can be achieved by growing micro-cuttings on pretreatment media containing cytokinin, antioxidants, and elicitors of defense proteins for 2–3 weeks, so one harvests relatively homogeneous 1 mm apical shoot tips. The addition of exogenous antioxidants and elicitors of defense proteins during preculturing helps improve the regrowth efficiency and quality of the plantlets of cryopreserved *Vitis* shoot tips [16,35,36,37,76,102].

Since the first report on grapevine shoot tip cryopreservation was published in 1989, much research has been carried out, with methods that been optimized to achieve post-thaw regrowth levels that satisfy the genebank standards for the implementation of cryopreserved *Vitis* collections. Reports on grapevine shoot tip cryopreservation using droplet-vitrification procedures that are applicable to a wide range of *Vitis* species [35,36,37] show promise for overcoming genotype-specific responses. Finally, the first effort to implement a cryopreserved *Vitis* collection has been initiated at USDA ARS National Laboratory for Genetic Resources Preservation, in Fort Collins, Colorado. At the present time, 28 *Vitis* accessions have been cryopreserved and stored within the cryotanks in LNV (Volk, personal communication).

Developing multiple cryopreservation methods for the successful preservation of a given plant species may help overcome genotype-specific responses; if one protocol fails, another may be successful for the same genotype within the species [29,46]. The V cryo-plate method uses shoot tips adhered to cryo-plates to facilitate manipulation, reduce mechanical injury from handling, and loss of shoot tips during the course of the cryopreservation protocol. This method follows the same principles as the droplet-vitrification method, by using ultra-fast shoot tip cooling and warming rates, an important requirement for successful cryopreservation protocols based on vitrification [147]. This method resulted in high regrowth levels, with high-quality plants obtained from cryo-exposed shoot tips, making it a practical and promising *Vitis* cryopreservation methodology [76]. Further work to determine the applicability of the V cryo-plate method across additional *Vitis* species would be advantageous.

Recent advances in *Vitis* cryopreservation research showed that shoot tips excised directly from plants that were not in tissue culture could be cryopreserved by droplet-vitrification [102,103]. This would increase the efficiency of cryoprocessing in genebanks by significantly reducing the laborious steps of in vitro culture establishment and multiplication. The applicability of this protocol, using alternative sources of explants (particularly those originating from the field), to additional *Vitis* accessions needs to be determined.

*Vitis* is highly susceptible to virus infections. The availability of cryopreservation protocols developed for long-term *Vitis* preservation may also facilitate the use of cryotherapy methods in the production of virus-free plants. To date, studies on grapevine have shown the effectiveness of cryotherapy in the elimination of GVA, GFLV, GLRaV-1, GLRaV-2, and GLRaV-3.

This review provides comprehensive information on the development and recent progress of *Vitis* shoot tip cryopreservation and cryotherapy that resulted in healthy plants with high regrowth levels across diverse *Vitis* species. The next challenges for *Vitis* cryopreservation involve finding strategies to minimize labor inputs within cryo-banks, and to identify and prioritize collections to ensure the long-term security of *Vitis* genebank materials.

## Figures and Tables

**Table 2:** Shoot tip cryotherapy for eradication of grapevine (*Vitis* spp.) viruses.

Specie and Genotype	Virus	Cryo Method	Explant	Regrowth (%)	Plant Conditions during Virus Detection	Virus-Free Frequency (%)	Virus Confirmation Method	Year Ref.
*V. vinifera* ‘Bruti’	GVA	VI	ST (1 mm; type n/s)	50	Plants grown in the greenhouse for 4 months	97	Western blotting/ELISA	2003 [64]
		ED		62				
*V. vinifera* ‘Black’	GVA	ED	ST (1 mm; type n/s)	59	In vitro plants that are 2 to 4 months old	42.2	RT-PCR	2011 [63]
*V. vinifera* Nebbiolo	GVA GLRaV-3	EV	AST (2 mm)	15	n/s	100	Multiplex RT-PCR	2012 [167]
*V. vinifera* Chardonnay	GFLV	DV	AST (1 mm)	30.7	In vitro plants that are 2 months old	77.8	ELISA	2015 [34]
*V. vinifera* Cabernet Sauvignon	GLRaV-3			41.6		100		
*V. vinifera* Pinot gris	GLRaV-2	DV	AST and AxST (size n/s)	13.6	In vitro plants that are 3 months old/plants grown in the greenhouse for 3 and 6 months	100	DAS-ELISA	2015 [62]
*V. vinifera* Sauvignon blanc 316	GLRaV-2			15.7		100 and 100		
*V. vinifera* Sauvignon blanc	GLRaV-1 and GLRaV-3			30		100		
*V. vinifera* ‘Lakemont Seedless’	GLRaV-3			16.2		100		
*V. vinifera* Chardonnay	GLRaV-3			13		100		
*V. vinifera* Cabernet Sauvignon	GLRaV-3	DV	AST (1 mm)	59	Plants grown in the screen-house for 12 months	100	RT-PCR and MD RT-PCR	2018 [51]
*V. vinifera* Chardonnay				47				
*V. vinifera* × *V. labrusca* ‘Kyoho’				51				
*V. pseudoreticulata* ‘Hunan-1’				43				

GVA: *Grapevine virus A*; GFLV: *Grapevine fanleaf virus*; GLRaV-3: *Grapevine leafroll-associated virus-3*; GLRaV-2: *Grapevine leafroll-associated virus-2*; GLRaV-1: *Grapevine leafroll-associated virus-1*; ST: shoot tips; AxST: axillary shoot tips; AST: apical shoot tips; VI: vitrification; ED: encapsulation-dehydration; EV: encapsulation-vitrification; DV: droplet-vitrification; RT-PCR: double-antibody sandwich enzyme-linked immunosorbent assay; MD RT-PCR: microtissue direct transcription polymerase chain reaction; ELISA: enzyme-linked immunosorbent assay; DAS-ELISA: double-antibody sandwich enzyme-linked immunosorbent assay; n/s: not specified.

## Abbreviations

AD	Apical dome
AsA	Ascorbic acid
AST	Apical shoot tips
AxST	Axillary shoot tips
BA	Benzylaminopurine
DAS-ELISA	Double-antibody sandwich enzyme-linked immunosorbent assay
DMSO	Dimethyl sulfoxide
ELISA	Enzyme-linked immunosorbent assay
FWB	Fresh weight basis
IHC	Immunohistochemical
LN	Liquid nitrogen
LNV	Liquid nitrogen vapor
LS	Loading solution
ULS	Unloading solution
SA	Salicylic acid
GVA	*Grapevine virus A*
GFKV	*Grapevine fleck virus*
GFLV	*Grapevine fanleaf virus*
GLRaV-1	*Grapevine leafroll-associated virus-1*
GLRaV-2	*Grapevine leafroll-associated virus-2*
GLRaV-3	*Grapevine leafroll-associated virus-3*
GSH	Glutathione
LP	Leaf primordia
MD RT-PCR	Microtissue direct reverse transcription polymerase chain reaction
MS	Murashige and Skoog (1962) medium
NAA	Naphthaleneacetic acid
PPV	*Plum pox virus*
PVS	Plant vitrification solution
PVS2	Plant vitrification solution 2
PVS3	Plant vitrification solution 3
RT-PCR	Reverse transcription polymerase chain reaction
ROS	Reactive oxygen species
ST	Shoot tips
V cryo-plate	Vitrification cryo-plate
ZR	Zeatin riboside
